# Mesoporous Materials and Nanoscale Phenomena in Hybrid Photovoltaics

**DOI:** 10.3390/nano12081307

**Published:** 2022-04-11

**Authors:** Alessandra Alberti

**Affiliations:** Consiglio Nazionale delle Ricerche-Istituto per la Microelettronica e Microsistemi (CNR-IMM), 95121 Catania, Italy; alessandra.alberti@imm.cnr.it

Hybrid photovoltaics (H-PV), initiated as dye-sensitized solar cells (DSC) by prof. Michael Graetzel (EPFL Lausanne, Switzerland) and subsequentially remodulated as perovskite solar cells (PSCs) by prof Tsutomu Miyasaka (Toin University of Yokohama, Japan), are revolutionizing the views to satisfy the increasing demand for renewable energy. The photon-to-current efficiency record of PSC is remarkably high and close to the record of silicon-based solar cells, with single-crystal silicon devices and PSC currently at 26.1% and 25.7%, respectively (NREL chart, 22-02-22). The technological breakthrough of PSCs stems, on the one hand, from a cost-efficiency convenience and, on the other hand, from the perspective of overcoming the single-cell Shockley–Queisser limit as they are combined in tandem architectures [1]. Together with outdoor PV, dedicated hybrid solar cell architectures for indoor powering [2] and aerospace [3] are likewise in the spotlight. Different device schemes, various kinds of transporting layers and the versatile perovskite composition have constantly boosted the efficiency of PSCs. Easy compositional modifications accomplished by simple solution processing represent a huge strength to satisfy specific demands [4]. As recent advances in the field, vacuum deposition of Perovskites have progressively complemented chemical methods [5] likewise all-inorganic perovskite solar cells have made progress in terms of efficiency that targeted 19% record values [6,7].

In this highly challenging framework, materials science has been playing a major role with the pivotal involvement of mesoporous materials [8] and phenomena at the nanoscale [9,10] that take place in the core as well as at the multi-interfaces established in the layered architecture of DSC [11] and PSC [12,13].

This Special Issue of *Nanomaterials* welcomed contributions on demanding aspects in the growth/synthesis, functionalization, interfacing, light absorption, carrier generation and injection, stabilization, and integration of materials in DSC and PSC architectures, with a special focus on mesoscale behaviour and nanoscale phenomena.

In the picture from DSC to PSC, the list of materials investigated along the Special Issue includes metal oxides and molecular layers for carrier extraction, transparent and conductive oxides, photoactive molecules and additives, and photoactive perovskites. A “Green Route towards Stable Dye-Sensitized Solar Cells” is signed by a paper on 100% aqueous hydrogel electrolytes that are nontoxic and non-flammable in the pathway of the environmental sustainability for solar energy harvesting. Two papers focus on TiO*_x_* as an electron transporting layer for PSC, capturing the need to keep low the thermal budget for its production and highlighting the pivotal role of nano-structuring the layer for a better charge extraction. Coupled with facile nanoimprinted one-dimensional grating nanopatterning (1D GNP), an improved light utilization with consequent enhancement of the device performances is demonstrated for CsPbIBr_2_-based PSC. Non-standard materials such as CuSCN prepared by electrodeposition from an aqueous solution and CuCrO_2_ thin films to be implemented as p-type electrodes are explored in the Special Issue for architectures of solar devices.

The Special Issue also touches the hot topic of the structural stability of perovskites with a case study of FAPbI_3_ and MAPbI_3_. It is argued how the effect of interstitial water is progressively disruptive against the compactness of the perovskite structure by promoting orthorhombic delta or hydrated phases with loosely bonded chains of PbI_6_ octahedra. Another case study is provided by a work on the structural competition between the black and the yellow phases in CsPbI_3_ lattices. Since the competition is mainly piloted by thermodynamics, stabilization strategies are proposed consisting of either the confinement of the material into nano-sized structures or by integrating Europium atoms as host species into the perovskite lattice for multi-fold actions that freeze the black phase.

On the strategic role of material confinement, a paper in this Special Issue deals with the inclusion of 2D transition metal dichalcogenides in perovskite inks and shows their influence on solar cell performance. The view embraces from single to multi-cations perovskites applied to state-of-the-art solar cells demonstrators. Throughout this Special Issue, as in this last case, the efficiency of proof-of-concept devices was settled over 15%.

Active interfaces, defects, lattice structures, low dimensionality and the operational behavior of advanced materials for DSC and PSC devices were indeed of relevance for this Special Issue to increase knowledge and awareness on some challenges still debated in the research community.
